# Hypersarcosinemia presenting as acute leukoencephalopathy with restricted diffusion in a young child

**DOI:** 10.1186/s12887-026-07217-3

**Published:** 2026-06-23

**Authors:** Jia Shi, Chunhua Zhang, Shuhong Ren, Weihua Zhang, Xiuwei Zhuo, Changhong Ding

**Affiliations:** 1 Department of Neurology, Baoding Hospital of Beijing Children’s Hospital,Capital Medical University, Baoding, 071000 China; 2 Department of Neurology, Beijing Children’s Hospital,Capital Medical University,National Center for Children’s Health, Beijing, 100045 China; 3 Department of Research & Development, MILS International, Yokohama, 222-0033 Japan

**Keywords:** SARDH, Hypersarcosinemia, Aseptic encephalitis, Clinical imaging phenotypes, Case report

## Abstract

**Background:**

Hypersarcosinemia, resulting from sarcosine dehydrogenase (SARDH) gene mutation, is a rare autosomal recessive disorder with variable, often nonspecific clinical presentations, leading to diagnostic difficulty and low clinical awareness.

**Case presentation:**

A 6-year-old boy presented with clinical features suggestive of viral encephalitis, including headache, vomiting, intermittent fever, and lethargy. Initial MRI revealed cytotoxic edema in the subcortical white matter and splenium of the corpus callosum. Although symptoms improved transiently with steroid therapy, persistent imaging abnormalities prompted metabolic and genetic evaluations. Metabolic and genetic investigations confirmed a diagnosis of hypersarcosinemia, with markedly elevating sarcosine levels and compounding heterozygous SARDH mutations. Genetic testing identified compound heterozygous mutations in the SARDH gene (c.293G > C and c.679 C > T), confirming hypersarcosinemia. Following initiation of folic acid and mecobalamin, partial radiological improvement was observed, although a causal relationship could not be established.

**Conclusions:**

This case highlights the diagnostic challenge of hypersarcosinemia and its potential mimicry of acquired encephalitis. To our knowledge, this is the first report describing an acute encephalitis-like presentation accompanied by persistent cytotoxic edema on MRI, thereby suggesting a possible expansion of the known clinical and neuroimaging spectrum of this disorder, although this observation requires confirmation in additional cases. However, given the rarity of hypersarcosinemia and the possibility of underreporting, the absence of prior similar reports should be interpreted with caution. In this case, genetic testing was essential for establishing the diagnosis, although the necessity of genetic testing in all cases of unexplained white matter changes cannot be determined from a single report. The temporal association of partial radiological improvement with folic acid and mecobalamin supplementation is hypothesis-generating only and requires further investigation ; no causal or therapeutic conclusion can be drawn from this single case.

**Supplementary Information:**

The online version contains supplementary material available at https://doi.org/10.1186/s12887-026-07217-3.

## Background

Hypersarcosinemia, also referred to as sarcosine dehydrogenase (SARDH) deficiency, is a relatively rare autosomal recessive genetic disorder [1]. The clinical manifestations may include intellectual disability, epilepsy, myoclonus, ataxia, language disorder, and vision loss [2]. Due to SARDH deficiency, sarcosine accumulates in the plasma and its excretion in urine increases [2]. The condition is generally considered benign, as many individuals identified through screening remaining entirely asymptomatic. However, some case reports have described a spectrum of non-specific and variable neurological manifestations, including intellectual disability, developmental delay, epilepsy, ataxia, and speech disorders. Additionally, severe but controversial presentations involving progressive neurological deterioration, optic atrophy, and hypertrophic cardiomyopathy have been documented in isolated cases [2]. This report presents the clinical manifestations, cranial magnetic resonance imaging (MRI) features, and genetic characteristics of a child diagnosed with hypersarcosinemia, who initially exhibited symptoms resembling viral encephalitis.

## Case presentation

### Initial illness and management at a local hospital (December 2024)

In December 2024, the previously healthy 6-year-old boy first presented to a local tertiary care hospital with a 1-month history of headache, dizziness, vomiting, and intermittent fever. He was hospitalized there for 14 days. The cerebrospinal fluid (CSF) analysis is detailed in Table 1. Initial brain MRI and electroencephalogram were normal. A provisional diagnosis of viral encephalitis was made. His symptoms improved after a 9-day course of acyclovir, mannitol, and methylprednisolone, and he was discharged.

**Table 1:** Comprehensive Cerebrospinal Fluid (CSF) analysis

Parameter	Result
Appearance	Clear and colorless
White Blood Cell (WBC) count	73 × 10⁶/L
Differential count	Mononuclear cells predominant
Glucose	2.89 mmol/L
Total Protein	314.4 mg/L
Chloride	127.1 mmol/L
Adenosine deaminase	0.2 U/L
Lactic acid	1.44 mmol/L

### Second episode and re-evaluation

Three days prior to admission at our center, the patient’s headache and vomiting recurred, accompanied by lethargy. He was consequently admitted to our Department of Neurology on January 10, 2025 (designated as Day 1 of the current admission for clarity in describing subsequent events). Neurological examination was notable only for a possible right Babinski sign; meningeal signs were negative.

### Diagnostic investigations and imaging evolution

Relevant laboratory investigations revealed CSF pleocytosis (73 × 10^6^/L) with elevated interleukin-6 (526.26 pg/mL). Serum myelin oligodendrocyte glycoprotein (MOG) antibody was weakly positive at a titer of 1:10. Extensive tests for infectious, autoimmune, and paraneoplastic etiologies were negative.

**Fig. 1:**
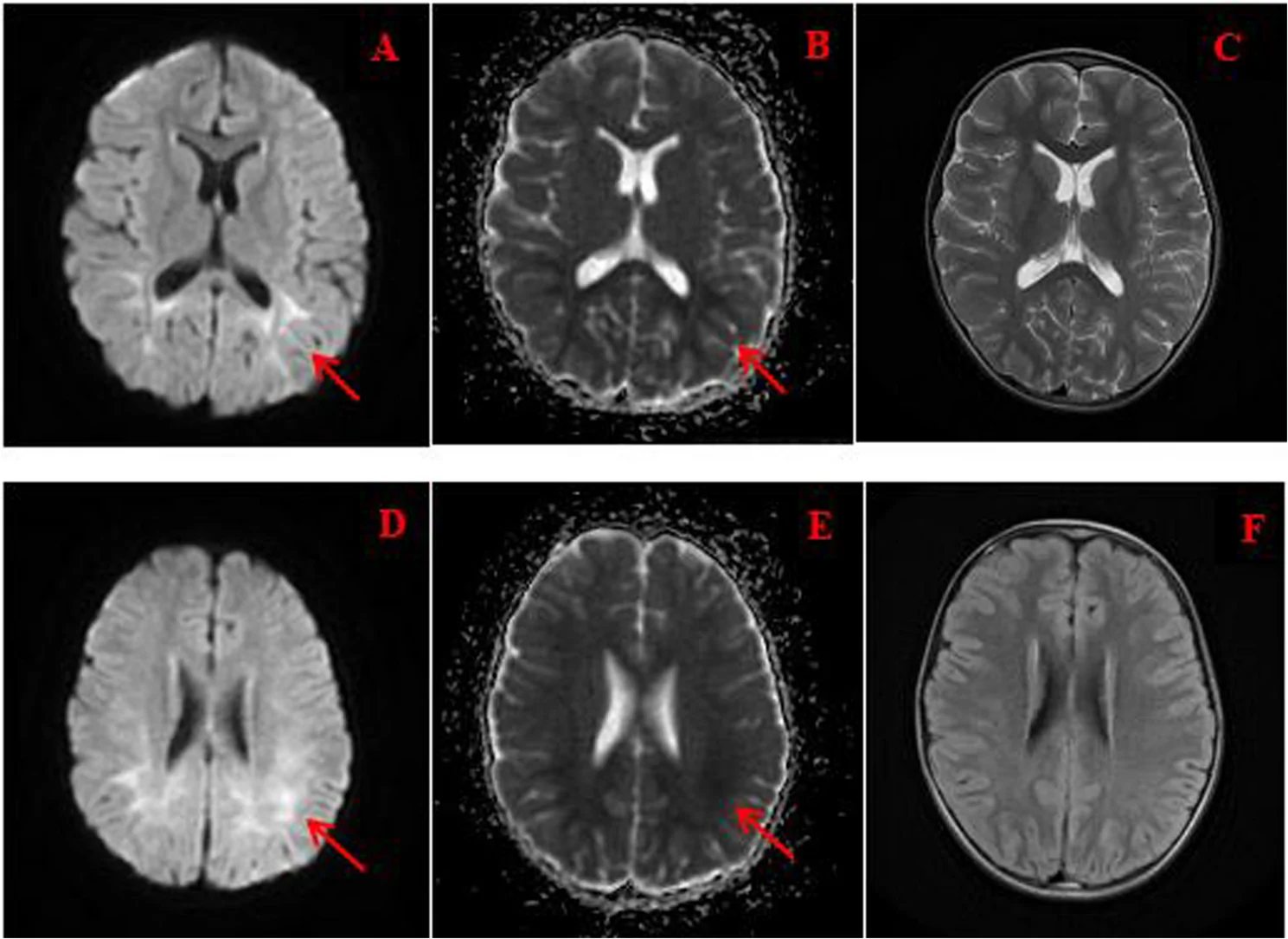
Brain MRI results of the child with hypersarcosinemia (January 12, 2025). **A** and **D** Patchy DWI hyperintense signal in the subcortical white matter of the occipital lobe of the bilateral cerebral hemispheres (arrows). **B** and **E** Patchy hypointense signal on ADC maps in the subcortical white matter of the occipital lobe of the bilateral cerebral hemispheres (arrows). **C** The T2-weighted image showed no abnormalities. **F** The FLAIR image showed no abnormalities. MRI, Magnetic resonance imaging. DWI, Diffusion-weighted imaging. ADC, Apparent diffusion coefficient. FLAIR, Fluid-attenuated inversion recovery

Brain MRI at admission revealed cytotoxic edema (restricted diffusion on DWI/ADC sequences, meaning bright on DWI and dark on ADC, indicative of cellular swelling ) in the subcortical white matter and splenium of the corpus callosum (Fig. 1). A working diagnosis of aseptic encephalitis was considered. He received intravenous methylprednisolone pulse therapy followed by an oral taper, leading to rapid clinical resolution of symptoms within 5 days.

**Fig. 2:**
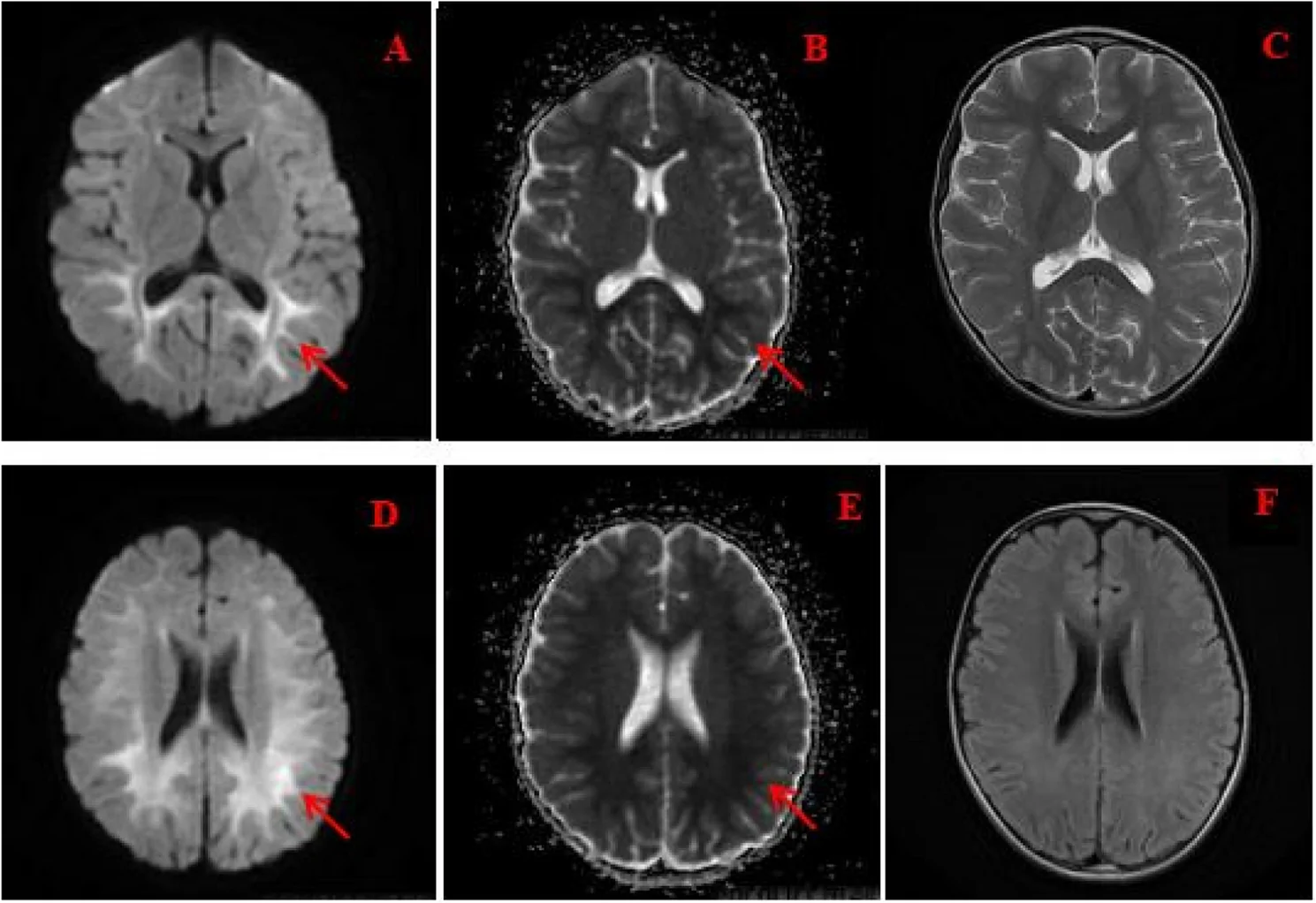
Brain MRI results of children with hypersarcosinemia(January 26, 2025, 14 days after admission and following clinical recovery). **A** and **D**: Large patches of DWI hyperintense signal in the subcortical white matter of the bilateral cerebral hemispheres were observed, and the range was larger than before (arrows). **B** and **E**: Large patches of subcortical white matter in the bilateral cerebral hemispheres showed hypointense on ADC maps (arrows). **C**: The T2-weighted image showed no abnormalities. **F**: The FLAIR image showed no abnormalities. MRI, magnetic resonance imaging. DWI, diffusion-weighted imaging. ADC, apparent diffusion coefficient. FLAIR, fluid-attenuated inversion recovery

**Fig. 3:**
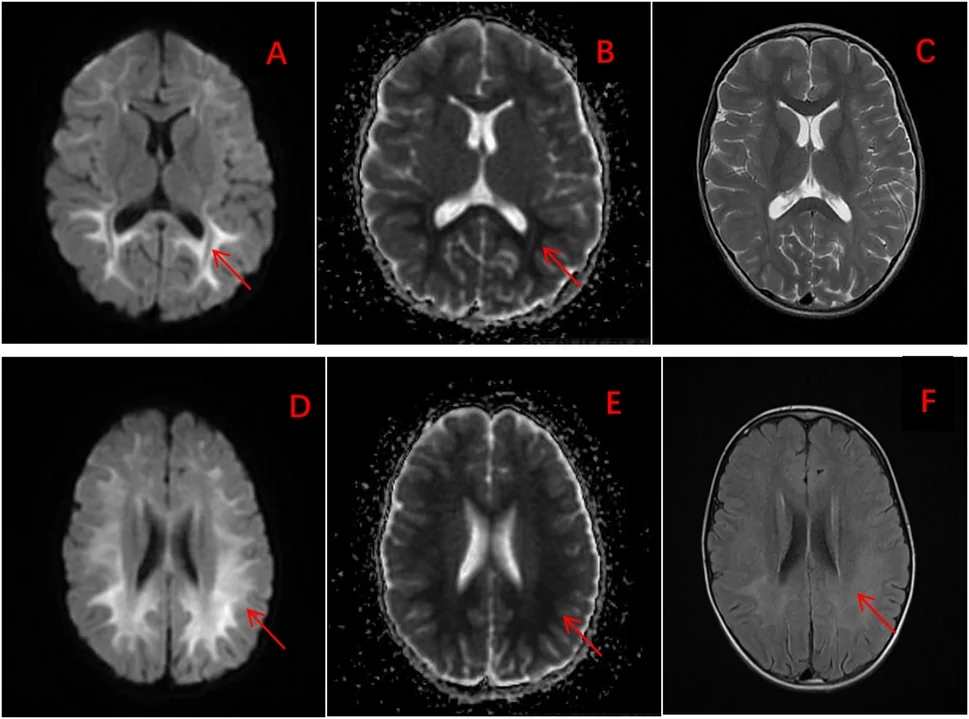
Brain MRI results of children with hypersarcosinemia (February 12, 2025, half a month after discharge).A and D: Large patches of DWI hyperintense signal in the subcortical white matter of the bilateral cerebral hemispheres (arrows). B and E: Large patches of subcortical white matter in the bilateral cerebral hemispheres showed hypointense on ADC maps (arrows). C: T2-weighted images showed no abnormalities. F: Slight FLAIR hyperintensity in the white matter of the posterior horn of the bilateral ventricles (arrows). MRI, magnetic resonance imaging. DWI, diffusion-weighted imaging. ADC, apparent diffusion coefficient. FLAIR, fluid-attenuated inversion recovery.

**Fig. 4:**
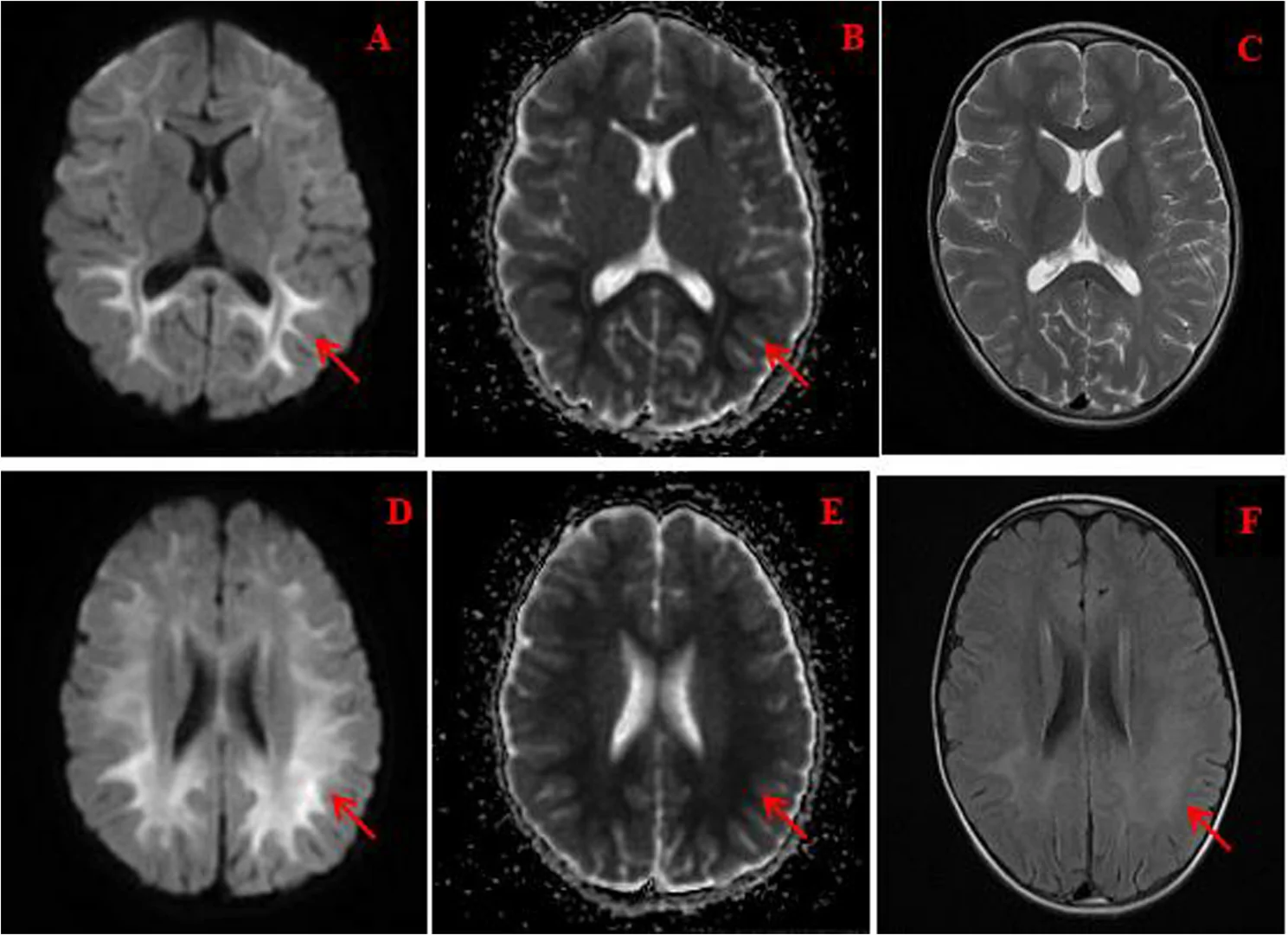
Brain MRI results of children with hypersarcosinemia (March 12, 2025, one and a half months after discharge). **A** and **D**: Large patches of DWI hyperintense signal in the subcortical white matter of the bilateral cerebral hemispheres (arrows). **B** and **E**: Large patches of subcortical white matter in the bilateral cerebral hemispheres showed hypointense on ADC maps (arrows). **C**: T2-weighted images showed no abnormalities. **F**: Slight FLAIR hyperintensity in the white matter of the posterior horn of the bilateral ventricles (arrows). MRI, magnetic resonance imaging. DWI, diffusion-weighted imaging. ADC, apparent diffusion coefficient. FLAIR, fluid-attenuated inversion recovery

**Fig. 5:**
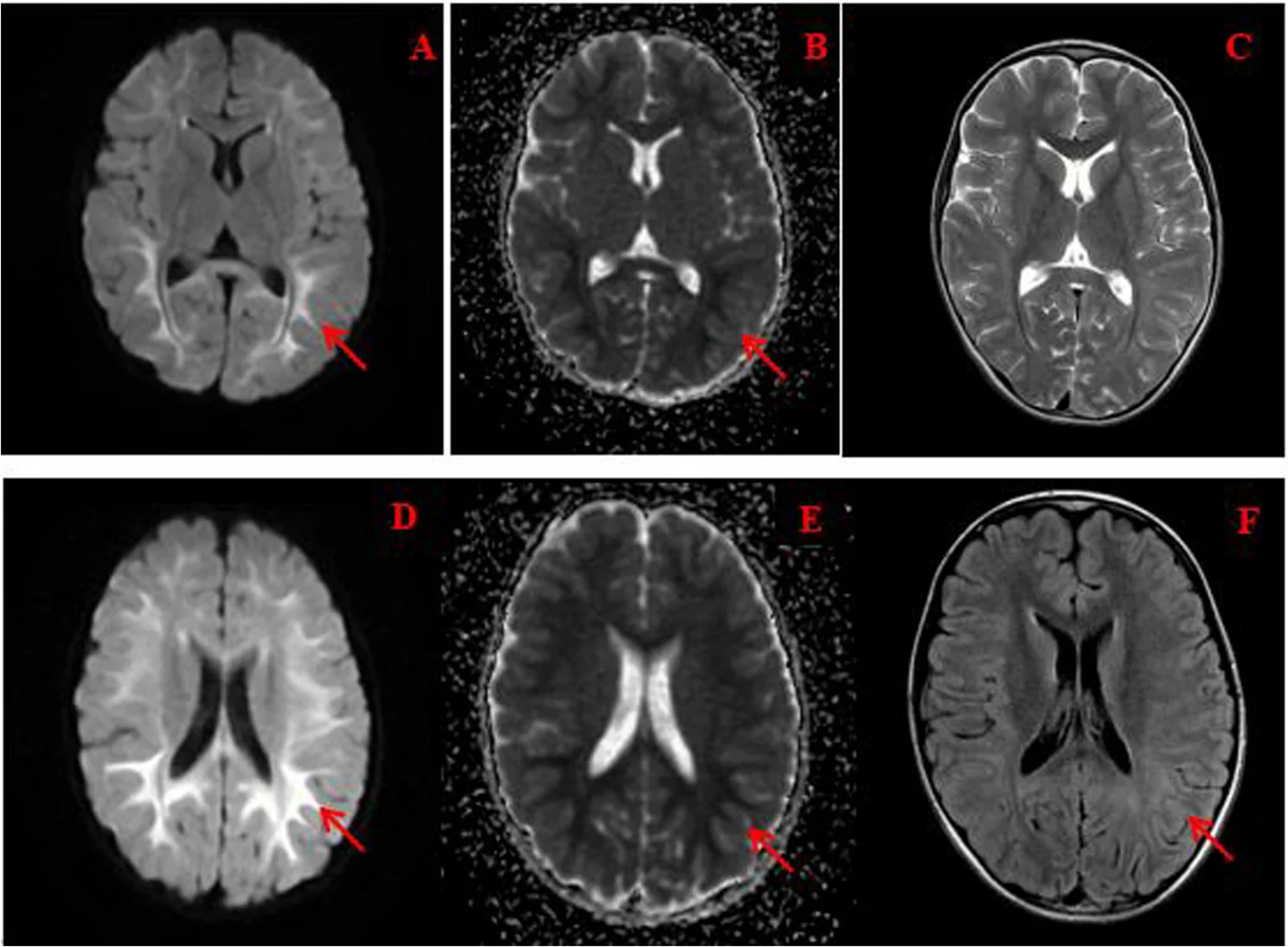
Brain MRI results of children with hypersarcosinemia (April 18, 2025, two and a half months after discharge). **A** and **D**: Large patches of DWI hyperintense signal in the subcortical white matter of the bilateral cerebral hemispheres (arrows). **B** and **E**: Large patches of subcortical white matter in the bilateral cerebral hemispheres showed hypointense on ADC maps (arrows). **C**: T2-weighted images showed no abnormalities. **F**: Slight FLAIR hyperintensity in the white matter of the posterior horn of the bilateral ventricles (arrows). MRI, magnetic resonance imaging. DWI, diffusion-weighted imaging. ADC, apparent diffusion coefficient. FLAIR, fluid-attenuated inversion recovery

However, follow-up MRI 14 days later showed marked progression of the cytotoxic edema (Fig. 2). Crucially, subsequent MRIs over 2.5 months demonstrated persistent, unchanged areas of restricted diffusion (Figs. 3, 4 and 5), establishing a pattern of prolonged cytotoxic injury. This dissociation between clinical improvement and persistently abnormal imaging raised strong suspicion for an underlying inherited metabolic leukoencephalopathy.

### Treatment and follow-up

**Fig. 6:**
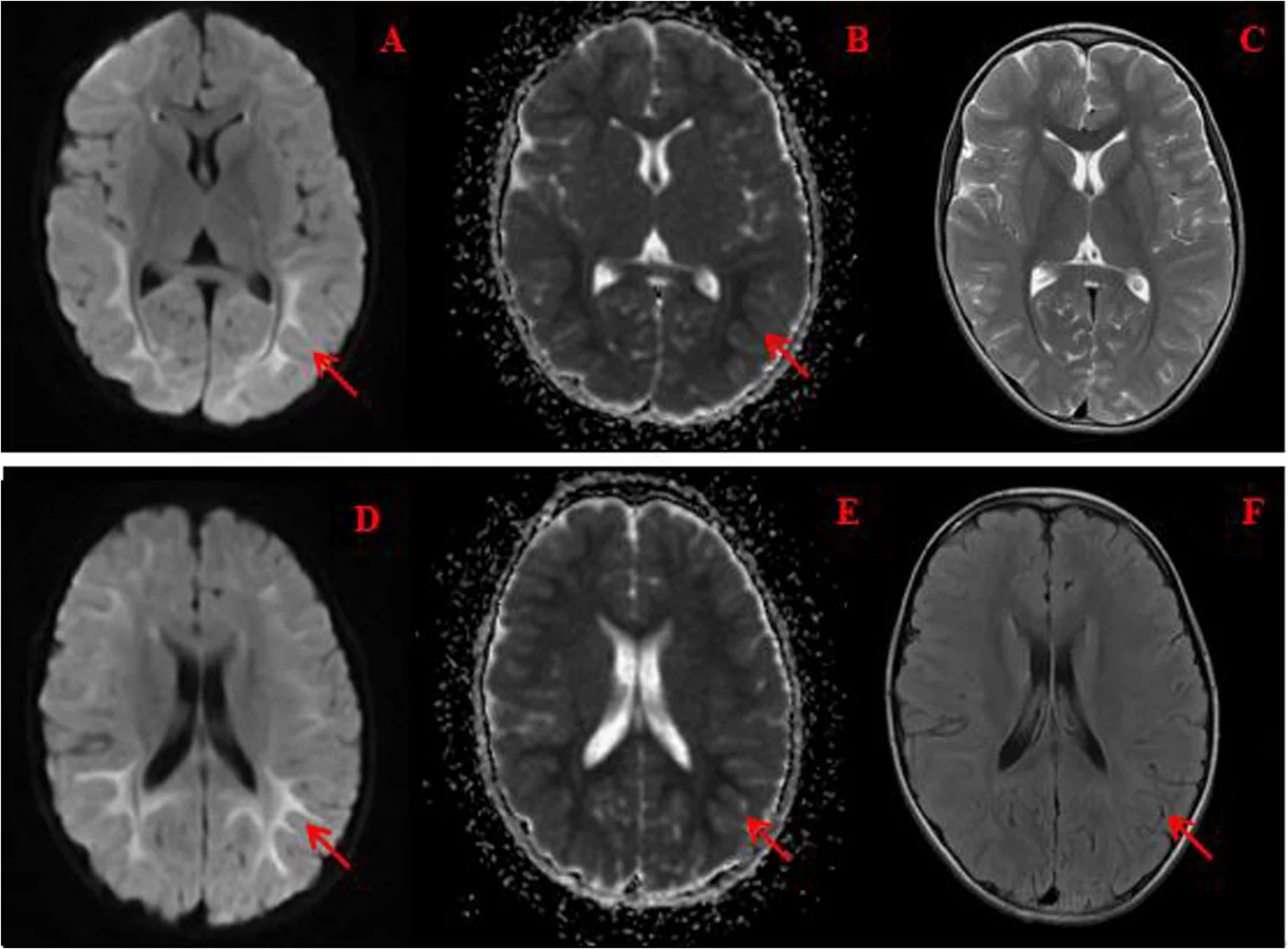
Brain MRI results of the child with hypersarcosinemia (June 13, 2025, four and a half months after discharge). **A** and **D**: DWI hyperintense signal in the subcortical white matter of the bilateral cerebral hemispheres was observed, and the degree of intensity increase was slightly reduced (arrows). **B** and **E**: Hypointense signals on ADC maps in the subcortical white matter of the bilateral cerebral hemispheres (arrows). **C**: The T2-weighted image showed no abnormalities. **F**: Slight FLAIR hyperintensity in the white matter of the posterior horn of the bilateral ventricles (arrow). MRI, magnetic resonance imaging. DWI, diffusion-weighted imaging. ADC, apparent diffusion coefficient. FLAIR, fluid-attenuated inversion recovery

Based on the role of folate in one-carbon metabolism, a therapeutic trial with oral folic acid (5 mg/day) and mecobalamin (0.5 mg/day) was initiated three months post-discharge. A follow-up MRI 1.5 months later (4.5 months post-discharge) showed partial reduction in the DWI abnormalities (Fig. 6). Concurrent urine metabolic screening demonstrated a significant decrease in sarcosine excretion. The patient remained clinically asymptomatic during this period.

### Screening for metabolic defects

**Fig. 7:**
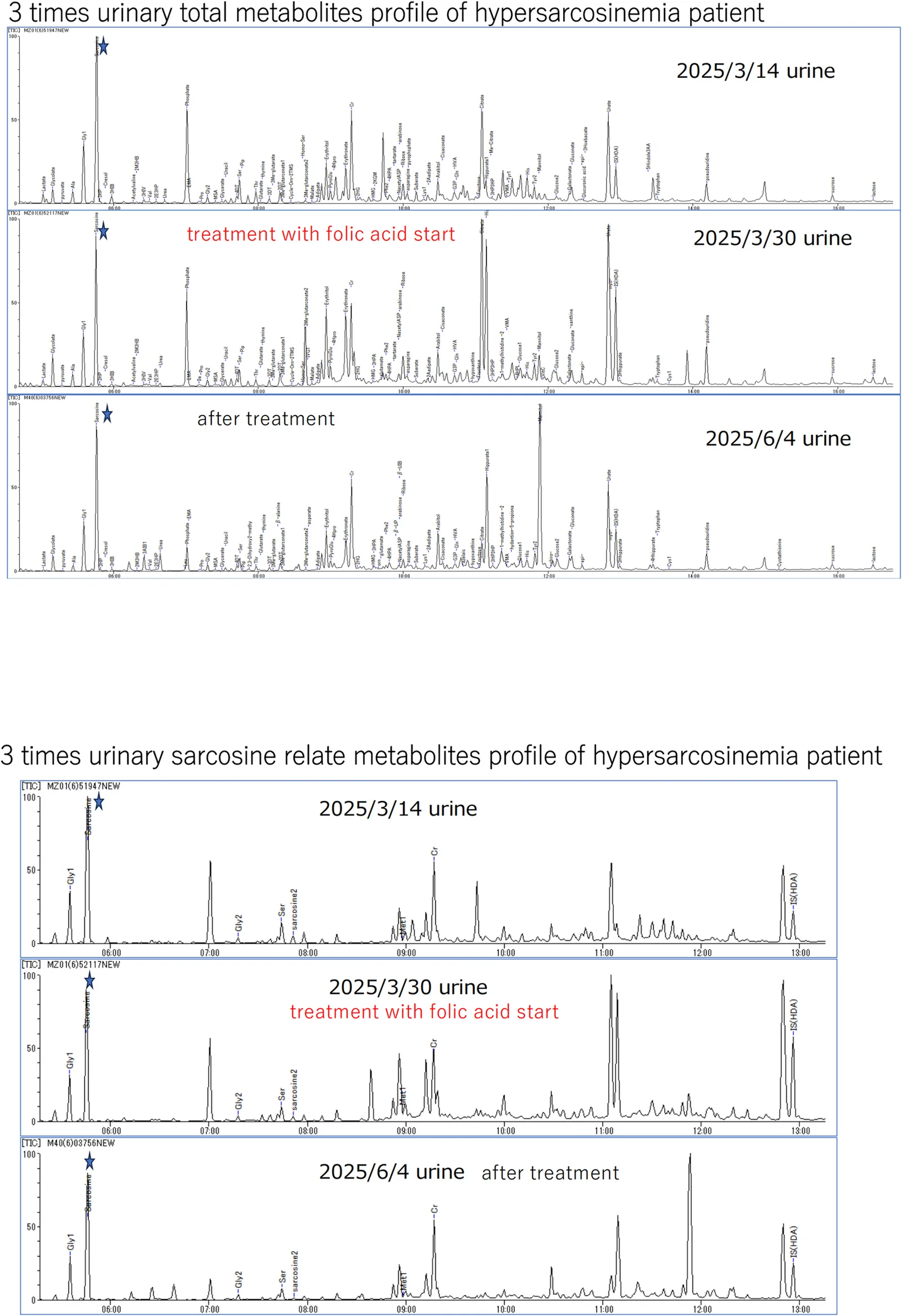
Comparison of three urine metabolic screenings and urine sarcosine values

The results of dry blood spot and urine metabolic screening initially indicated abnormalities in the ratio of propionyl-carnitine(C3) to acetyl-carnitine (C2) (C3/C2) and C24:0 lysophosphatidylcholines (LPC) (C24:0-LPC) indicators, accompanied by mild dicarboxylic aciduria and a significantly elevated level of sarcosine. Specifically, the blood levels of alanine(Ala), ratio of ornithine to citrulline (Orn/CIT), ratio of propionyl-carnitine(C3) to free-carnitine (C0)(C3/C0), C3/C2, C24:0-LPC, ratio of C24:0-LPC to C20:0-LPC (C24/C20), and C24:0-LPC to C22:0-LPC (C24/C22) were significantly increased, and there was also a marked increase in the level of sarcosine in urine. After 16 days, the metabolic screening results showed further significant increases in the levels of Ala, ratio of argine to ornithine (Arg/Orn), C0/C2, C3/C2 in blood, as well as in the sarcosine level in urine. After 66 days, the metabolic screening results revealed more than 70 times continued increase in the sarcosine content in urine than normal control (Fig. 7). A cerebrospinal fluid (CSF) metabolic screening review conducted four months later revealed elevated levels of sarcosine and glutamine, which were 5.95 times and 5.97 times higher, respectively, than those found in normal controls.

### Genetic testing

**Fig. 8:**
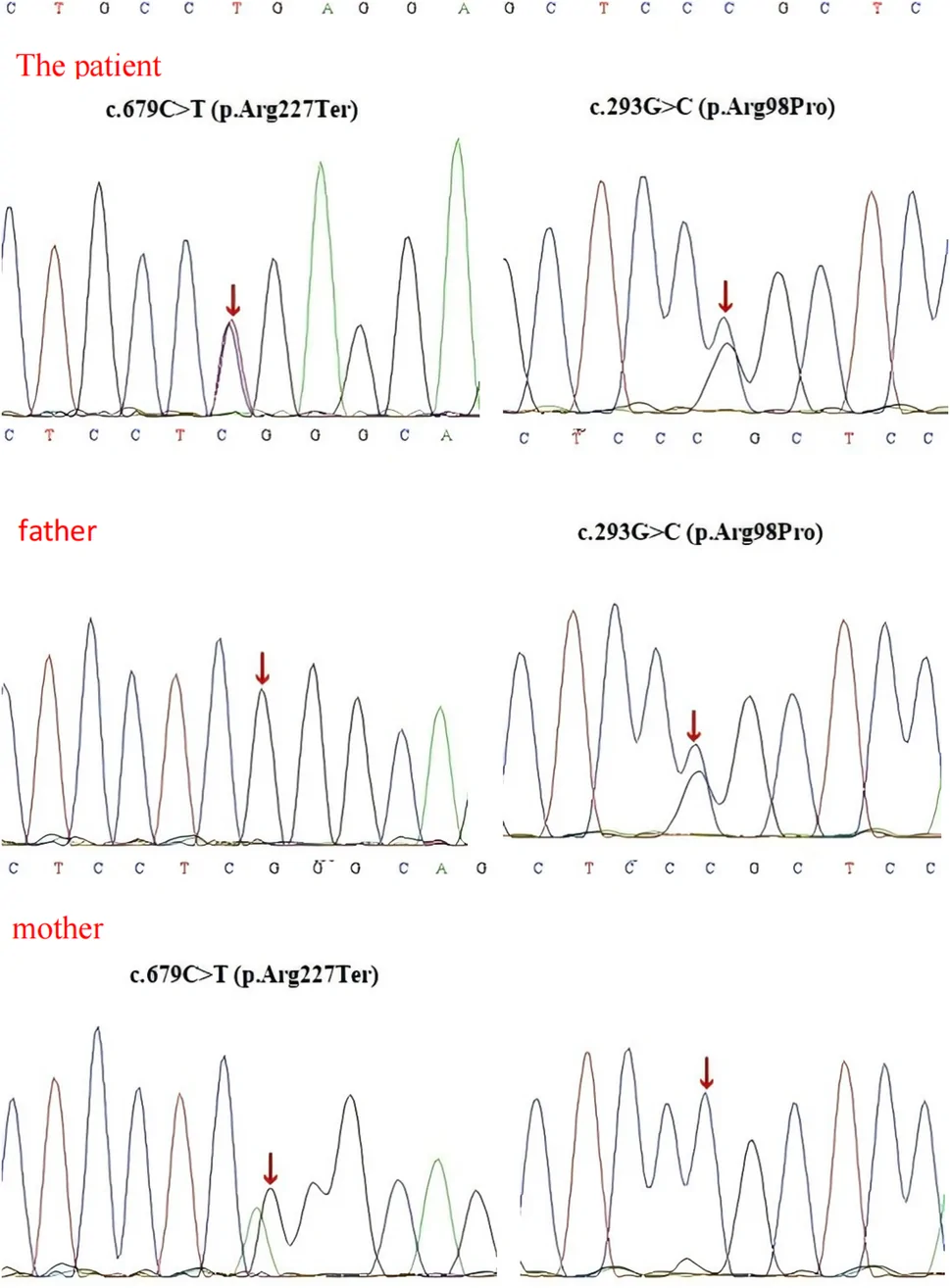
SARDH gene sequencing map of the patient and his parents

After obtaining informed consent from the patient and his parents, family exome sequencing was performed. It was found that the patient’s SARDH gene had compound heterozygous mutations (Fig. 8), specifically c.293G > C (p. Arg98Pro), which was inherited from his father; and c.679 C > T (p. Arg227Ter), which was inherited from his mother. According to the American College of Medical Genetics and Genomics criteria and Guidelines for the Classification of Genetic Variants, this variant was preliminarily identified as a suspected pathogenic variant. Based on the metabolic screening results, the patient was diagnosed with hypersarcosinemia.

## Discussion

Typically, sarcosine (N-methylglycine) is involved in the synthesis and decomposition of glycine within the one-carbon cycle pathway [3]. Sarcosine is mainly produced by the methylation of glycine. Within the mitochondrial matrix, sarcosine is metabolized into glycine and formaldehyde by enzymes like SARDH, and this metabolic pathway is maintained in the body. Under normal conditions, detecting sarcosine in human blood or urine is challenging. Deficiency of SARDH can result in the accumulation of sarcosine in the body, leading to hypersarcosinemia. Sarcosine accumulation can disrupt the balance between glycine and other one-carbon metabolites [4], thereby influencing the synthesis of biological macromolecules such as DNA and RNA, as well as normal cellular metabolism, ultimately resulting in cellular dysfunction and tissue and organ damage. Simultaneously, the accumulation of sarcosine may also exert toxic effects on the nervous system, influencing neurotransmitter metabolism and neural signal transmission, leading to the development of nervous system symptoms [5]. Gerritsen and Waisman reported the first case of hypersarcosinemia in 1966.

Hypersarcosinemia has an insidious onset and can manifest from infancy to adulthood [4]. Its clinical manifestations are diverse and lack specificity, including intellectual disability, epilepsy, myoclonus, etc. However, reports also indicate that many cases of hypersarcosinemia identified through newborn screening programs have normal phenotypes, suggesting individual variations in clinical symptoms. Mild cases may have delayed or even no clinical symptoms, usually diagnosed by incidental detection of elevated sarcosine in blood and urine during relevant examinations. This condition is thought to be associated with a mild reduction in enzyme activity, resulting in relatively mild accumulation of sarcosine in the body without significantly affecting the functions of tissues and organs [6]. At disease onset, the patient presented with headache, vomiting, impaired mental status and elevated CSF cell count, leading to a preliminary diagnosis of aseptic meningitis. The serum MOG antibody titer was 1:10, suggesting potential MOG antibody-associated disease; however, the low titer and atypical brain imaging findings failed to support this diagnosis. After glucocorticoid and symptomatic treatment, clinical symptoms alleviated, yet brain MRI showed progressive lesion expansion from early occipital white matter to the anterior cerebrum, manifested as prolonged symmetrical cytotoxic edema. These features prompted suspicion of inherited metabolic disorders, and subsequent blood-urine metabolic screening plus genetic testing confirmed the final diagnosis. It was inferred that infection-or immune-triggered metabolic disturbance caused sarcosine accumulation and subsequent neurotoxicity. Cerebellar vermis atrophy has been documented in previous case reports, while this acute onset with progressive persistent cytotoxic white matter edemahas not been explicitly reported in the available literature (though underreporting is possible), potentially broadening the clinical and neuroimaging spectrum of the disease. No obvious developmental delay was noted in this patient, though cranial imaging showed typical cytotoxic changes. This indicates hypersarcosinemia can exert central neurotoxicity, warranting further long‑term pathological observation.

At present, only one case has been reported in China, which was reported by the Ren Xiaotun team from Beijing Children’s Hospital [7]. The only previously reported Chinese case (Ren et al.) presented with intellectual disability and mutism, without restricted diffusion on MRI.

The clinical and imaging presentation of our case distinguishes it from previously reported instances of hypersarcosinemia, highlighting features that appear to be uncommon or previously undescribed in the limited literature (Table 2). Prior reports have primarily associated the condition with non-acute, chronic neurological manifestations such as developmental delay, intellectual disability, or cerebellar atrophy [5, 7, 8]. In contrast, our patient presented acutely with encephalitis-like features (fever, headache, CSF pleocytosis), leading to an initial misdiagnosis. Radiologically, whereas earlier cases often described normal imaging or non-specific atrophy, a distinctive feature observed in this case was the persistent and symmetrical cytotoxic edema in the cerebral white matter and corpus callosum, vividly captured on DWI/ADC sequences over several months. This pattern is highly atypical for typical inflammatory demyelination and served as an important clue towards a metabolic etiology. Furthermore, the observed radiological improvement and reduction in sarcosinuria following supplementation with folic acid and mecobalamin provide preliminary, yet valuable, clinical insight into a potential therapeutic strategy, which has been scarcely documented.

**Table 2:** Comparison of key features between previously reported cases of hypersarcosinemia and the present case

Feature	Typical Presentation in Prior Literature	Present Case
Onset	Insidious, chronic (infancy to adulthood)	Acute, encephalitic
Leading Symptoms	Developmental delay, intellectual disability, seizures, ataxia	Headache, vomiting, fever, lethargy (mimicking viral encephalitis)
Key MRI Findings	Often normal; isolated reports of cerebellar vermis atrophy	Persistent, symmetrical cytotoxic edema in subcortical white matter & corpus callosum (DWI/ADC hyper/hypointense)
CSF Profile	Rarely described	Lymphocytic pleocytosis, elevated IL-6
Response to Steroids	Not reported	Clinical symptoms improved
Long-term Imaging Evolution	Static or progressive atrophy	Slow resolution of cytotoxic edema over months
Reported Therapeutic Response	Limited data; no consistent benefit from memantine	Radiological and biochemical improvement with folate/mecobalamin

Comparisons are based on limited published literature (hypersarcosinemia is rare) and should not be considered representative of all cases

We interpret the acute episode as a primary metabolic decompensation masquerading as aseptic encephalitis. The persistent cytotoxic edema on MRI, may represent a hallmark of cellular energy failure, is consistent with a metabolic etiology. We hypothesize that an intercurrent, possibly subclinical, infection or immune trigger (as suggested by the CSF pleocytosis and elevated IL-6) precipitated an acute surge in sarcosine accumulation in this genetically predisposed child. We hypothesize that sarcosine neurotoxicity, potentially through disruption of one-carbon metabolism and mitochondrial function, may have contributed to widespread cytotoxic edema and secondary neuroinflammation. However, this mechanistic interpretation is speculative, as we did not perform direct measurements of mitochondrial function or neuronal metabolic status in this patient. This inflammatory response, evidenced by CSF lymphocytosis and elevated IL-6, thus mimicked the clinical and laboratory features of infectious encephalitis. Therefore, while the presentation phenocopied encephalitis, we propose that the fundamental process was metabolic, with inflammation as a downstream consequence rather than the primary driver. This speculative conceptual framework, if validated in future studies, would help reconcile the inflammatory CSF profile with the persistent cytotoxic imaging pattern ; however, at present it remains a hypothesis rather than a proven mechanism. Regardless, the clinical observation underscores the importance of considering inborn errors of metabolism in patients with unexplained encephalitis-like presentations.

The CSF profile in this case merits comparison with typical patterns observed in viral encephalitis and primary metabolic encephalopathies. In viral encephalitis, viral encephalitis typically shows lymphocytic pleocytosis and evidence of viral PCR [11]. Our patient’s CSF showed lymphocytic pleocytosis (73 × 10⁶/L) and elevated IL-6 (526.26 pg/mL), which initially supported an infectious or inflammatory etiology. However, extensive viral testing remained negative, and the clinical course diverged from typical viral encephalitis.

In contrast, most metabolic encephalopathies present with minimal or no CSF pleocytosis [12]. When CSF abnormalities occur in metabolic disorders, they more commonly reflect energy failure (elevated lactate), neurotransmitter disturbances, or specific metabolite accumulations rather than inflammatory cellular responses. The presence of significant lymphocytic pleocytosis in our case is therefore atypical for a pure metabolic encephalopathy.

We interpret the mixed CSF profile as potentially suggestive of secondary neuroinflammation triggered by metabolic stress, but we acknowledge significant uncertainty in this interpretation. The elevated sarcosine and glutamine levels confirm the underlying metabolic disturbance. However, the presence of CSF pleocytosis and elevated IL-6 could also reflect: (a) a coincidental, subclinical viral or bacterial infection that was not captured by routine pathogen testing; (b) a parallel, unrelated inflammatory process; or (c) a combination of metabolic stress and an intercurrent immune trigger. Without more definitive evidence—such as brain tissue histopathology, comprehensive metagenomic sequencing to exclude occult pathogens, or longitudinal cytokine profiling before, during, and after metabolic decompensation—we cannot confidently distinguish between these possibilities. Therefore, while we hypothesize that sterile neuroinflammation may have occurred, we emphasize that a coincidental or parallel inflammatory process cannot be excluded.

Hypothetical Mechanisms Underlying Cytotoxic Edema and CSF Pleocytosis. The patient’s distinctive neuroimaging phenotype of persistent cytotoxic edema enables a tentative mechanistic hypothesis, presented as a conceptual framework rather than definitive conclusions. Cytotoxic edema arises from intracellular water retention caused by impaired Na⁺/K⁺-ATPase activity and represents a key marker of cellular energy failure. We propose that sarcosine buildup in hypersarcosinemia may disrupt mitochondrial one-carbon metabolism. Such disruption could impair ATP production through direct mitochondrial toxicity or interference with the glycine cleavage system, leading to bioenergetic collapse that may underlie the MRI-observed cytotoxic edema.

Meanwhile, the CSF lymphocytic pleocytosis, though resembling infectious encephalitis, may reflect sterile neuroinflammation induced by metabolic stress. Excessive sarcosine or its metabolites may exert neurotoxic effects, activate microglia and astrocytes, and potentially trigger secondary blood-brain barrier disruption. These mechanisms remain unvalidated in the present case and are merely hypothesized to guide further research. Similar crosstalk between metabolic intoxication and innate immune activation has been reported in other inherited metabolic disorders, yet its relevance to hypersarcosinemia remains unclear.

The serial MRI findings in this case provided suggestive diagnostic clues for this patient. The initial and predominant radiological feature was cytotoxic edema, evidenced by persistent restricted diffusion (DWI hyperintensity/ADC hypotensity) in the subcortical white matter and splenium of the corpus callosum over several months (Figs. 1, 2, 3, 4 and 5). This pattern has been described as a recognized, though non-specific, feature of some metabolic leukoencephalopathies (e.g., mitochondrial disorders, certain aminoacidopathies), where it signifies sustained intracellular energy failure or toxic injury, as opposed to the vasogenic edema typical of acute inflammatory demyelination [9]. However, its presence in our patient does not imply that it is a universal or pathognomonic finding for hypersarcosinemia.

Three key observations from the imaging timeline argued against a primary inflammatory demyelinating process such as MOG antibody-associated disease (MOGAD): (1) Dissociation of clinical and radiological response: Despite rapid clinical improvement with steroids, the cytotoxic edema paradoxically progressed and then remained static for months. (2) Atypical radiological phenotype: The symmetrical, confluent, and persistent cytotoxic edema pattern is inconsistent with the typically asymmetric, large, and fluffy T2 lesions of MOGAD, which often show resolution on follow-up [10]. (3) Supporting serological context: The serum MOG antibody titer was very low (1:10). In this case, it is important to emphasize that such low-titer MOG antibodies (≤ 1:10) may be nonspecific and were therefore interpreted with caution; they should not be overinterpreted as diagnostic of MOGAD without typical clinical and radiological features. This observation, however, is derived from a single case and does not establish a general rule [11, 12]. In the setting of the above radiological mismatch and the lack of other supportive features (e.g., optic neuritis), this was considered more likely a false-positive or incidental finding rather than diagnostic of MOGAD. Therefore, the prolonged cytotoxic edema served as the pivotal clue that redirected the diagnostic workup from acquired neuroinflammation towards an inborn error of metabolism, ultimately leading to the diagnosis of hypersarcosinemia.

A critical observation in this case is the dissociation between clinical and radiological response to corticosteroid therapy. While the patient’s symptoms (headache, vomiting, lethargy) resolved rapidly following methylprednisolone treatment, serial MRIs demonstrated persistent and even progressive cytotoxic edema. This discordance carries an important diagnostic lesson: steroid responsiveness should not be interpreted as confirmation of an immune-mediated encephalitis. In clinical practice, rapid symptomatic improvement after corticosteroids is often viewed as supportive of an autoimmune or inflammatory etiology. However, as this case illustrates, steroids may transiently alleviate symptoms by reducing secondary inflammation, vasogenic edema, or blood-brain barrier permeability—without necessarily addressing the underlying metabolic defect or modifying the disease course. The persistent cytotoxic edema on DWI/ADC sequences, reflecting ongoing cellular energy failure, remained unchanged despite clinical recovery. This phenomenon represents a key diagnostic pitfall: reliance on clinical response to steroids can falsely reassure clinicians and delay consideration of neurometabolic disorders. Therefore, based on our experience with this single case, in some patients presenting with encephalitis-like symptoms, particularly when imaging reveals persistent cytotoxic edema, we suggest that the triad of (1) clinical improvement with steroids, (2) radiological persistence or progression, and (3) atypical imaging features should raise suspicion for an underlying inborn error of metabolism rather than a primary neuroinflammatory condition. However, the generalizability of this heuristic to other patients or other metabolic disorders remains unknown and requires validation in larger case series.

Evaluation of Folate and Mecobalamin Therapy. Currently, there is no established disease-modifying therapy for hypersarcosinemia. Our decision to initiate folate and mecobalamin was based on the central role of folate (as tetrahydrofolate) and vitamin B12 (as methylcobalamin, activated by mecobalamin) in the one-carbon metabolic cycle where sarcosine is metabolized. Supplementation aims to potentially enhance residual metabolic flux through alternative or parallel pathways, such as promoting the remethylation of homocysteine to methionine, thereby possibly reducing the precursor pool for sarcosine formation or facilitating its disposal. Following supplementation, we observed a reduction in urinary sarcosine excretion and partial radiological improvement. We reiterate that this temporal correlation does not establish causation. Spontaneous radiological improvement over time cannot be fully excluded, given the prolonged natural history of the condition and the lack of a control observation. The observed changes could equally reflect any of the following: (a) a true therapeutic effect of folate/mecobalamin; (b) gradual spontaneous resolution of the metabolic insult as part of the natural disease course; (c) regression to the mean; or (d) a combination of factors. We cannot distinguish among these possibilities. Therefore, this observation should be considered hypothesis-generating only rather than definitive proof of treatment efficacy. We explicitly caution against using this single case to support therapeutic decisions in future patients without further validation from larger studies or patient registries. It highlights a hypothetical potential therapeutic avenue—folate and B12 supplementation in disorders of one-carbon metabolism—that urgently requires validation through future studies, patient registries, or controlled clinical trials. We explicitly state that no treatment recommendation for hypersarcosinemia can be made based on this case. Clinicians should interpret these preliminary findings with caution and not assume universal treatment benefit without further evidence.

Genetic Variant Analysis and Pathogenicity. The diagnosis was confirmed genetically by the identification of compound heterozygous mutations in the SARDH gene: c.293G > C (p.Arg98Pro) and c.679 C > T (p.Arg227Ter). Their pathogenicity was assessed using the ACMG/AMP guidelines. The c.679 C > T variant is a nonsense mutation predicted to introduce a premature termination codon (p.Arg227Ter), leading to a truncated, non-functional protein. This fulfills the PVS1 (Very Strong) criterion. Its absence from population databases (gnomAD) supports the PM2 (Moderate) criterion. The c.293G > C variant is a missense change affecting a conserved residue. Its extreme rarity (PM2) and multiple lines of computational evidence (REVEL, SIFT, PolyPhen-2) predicting a deleterious effect support the PP3 (Supporting) criterion. Crucially, the patient’s highly specific biochemical phenotype of markedly elevated sarcosine provides PP4 (Supporting) evidence. The in trans configuration of these two variants perfectly explains the autosomal recessive inheritance and the severe biochemical perturbation, confirming the molecular diagnosis.

Limitations of generalizability from a single case report. As a single observational case report without a control group or confirmatory replication, this study has inherent limitations in generalizability. This single observational case cannot provide evidence for or against the efficacy of folic acid or mecobalamin in hypersarcosinemia. Spontaneous resolution, natural fluctuation, and regression to the mean remain equally plausible explanations for the observed temporal changes. Prospective studies or carefully designed patient registries are needed to address this question.

## Conclusion

In conclusion, based on this single case, this report suggests a possible expansion of the phenotypic spectrum of hypersarcosinemia to include an acute encephalitis-like presentation with prolonged cytotoxic edema on MRI, though confirmation in additional patients is needed. This presentation can lead to diagnostic confusion with acquired neuroinflammatory disorders. A low-tier MOG antibody result, in the context of atypical imaging, should be interpreted with caution. The dissociation between clinical and radiological response to steroids is a key red flag for an underlying neurometabolic condition. As a hypothesis to be tested in future studies, we propose that cytotoxic edema in this context could stem from sarcosine-induced bioenergetic failure. Direct evidence for this mechanism is lacking from our patient data. Furthermore, whether the observed CSF inflammation represented a primary, secondary, or coincidental process -- and whether steroid responsiveness reflected disease modification or non-specific symptomatic effects -- could not be determined from our data and remains an open question. The observed temporal association between initiation of folate/mecobalamin supplementation and subsequent biochemical/radiological changes is hypothesis-generating only. No causal conclusion can be drawn from a single observational case without a natural history comparator or pre-treatment baseline trajectory. Early metabolic screening for elevated sarcosine in cases of unexplained cytotoxic leukoencephalopathy is essential for timely diagnosis and genetic counseling.

## Supplementary Information

Supplementary Material 1.

## Data Availability

The datasets generated during and/or analyzed during the current study are available from the corresponding author upon reasonable request.
