# Supplementary material for: Hypersarcosinemia presenting as acute leukoencephalopathy with restricted diffusion in a young child
Source: BMC Pediatr. 2026 Jun 23;26:839. doi: 10.1186/s12887-026-07217-3 (PMC13551868; doi:10.1186/s12887-026-07217-3)
Supplement: Supplementary file 1 [file 12887_2026_7217_MOESM1_ESM.pdf]

CARE case report guidelines

CARE Checklist of information to include when writing a case report

(cc) BY-NC-ND

Topic	Item	Checklist item description	Reported on Line
Title	1	The diagnosis or intervention of primary focus followed by the words "case report"	Lines 2/Page 1
Key Words	2	2 to 5 key words that identify diagnoses or interventions in this case report, including "case report"	Lines 37/Page 2
Abstract (no references)	3a	Introduction: What is unique about this case and what does it add to the scientific literature?	Lines 31-36/Page 2
	3b	Main symptoms and/or important clinical findings	Lines 21-23/Page 2
	3c	The main diagnoses, therapeutic interventions, and outcomes	Lines 23-30/Page 2
	3d	Conclusion—What is the main "take-away" lesson(s) from this case?	Lines 31-36/Page 2
Introduction	4	One or two paragraphs summarizing why this case is unique (**may include references**)	Lines 40-46/Page 3
Patient Information	5a	De-identified patient specific information.	Lines49-51/Page 3
	5b	Primary concerns and symptoms of the patient.	Lines 51-54/Page 3
	5c	Medical, family, and psycho-social history including relevant genetic information	Lines 60-62/Page 3;Lines 63-68/Page 4
	5d	Relevant past interventions withoutcomes	Lines57-59/Page 4
Clinical Findings	6	Describe significant physical examination (PE) and important clinical findings.	Lines 17-22/Page 4
Timeline	7	Historical and current information from this episode of care organized as a timeline	Lines 52-62/Page 3
Diagnostic Assessment	8a	Diagnostic testing (such as PE, laboratory testing, imaging, surveys).	Lines 75-87 4; Lines 88-94/Page 5
	8b	Diagnostic challenges (such as access to testing, financial, or cultural)	Lines 172-183/Page 8
	8c	Diagnosis (including other diagnoses considered)	Lines 95-109/Page 5
	8d	Prognosis (such as staging in oncology) where applicable	Lines 115-118/Page 6
Therapeutic Intervention	9a	Types of therapeutic intervention (such as pharmacologic, surgical, preventive, self-care)	Lines 101/Page 5; Lines 116/Page 6
	9b	Administration of therapeutic intervention (such as dosage, strength, duration)	Lines 101-104/Page 5; Lines 116/Page 6
	9c	Changes in therapeutic intervention (with rationale)	Lines 116-118/Page 6
Follow-up and Outcomes	10a	Clinician and patient-assessed outcomes (if available)	Not Applicable
	10b	Important follow-up diagnostic and other test results	Lines 120-134/Page 6; Lines 136-143/Page 7
	10c	Intervention adherence and tolerability (How was this assessed?)	Lines209-213/Page 10
	10d	Adverse and unanticipated events	Not Applicable
Discussion	11a	A scientific discussion of the strengths AND limitations associated with this case report	Lines 191-197/Page 9
	11b	Discussion of the relevant medical literature **with references**.	Lines145-158/Page 7; Lines 159-170/Page 8
	11c	The scientific rationale for any conclusions (including assessment of possible causes)	Lines 206/Page 10
Patient Perspective	11d	The primary "take-away" lessons of this case report (without references) in a one paragraph conclusion	Lines 187-189/Page 9
Informed Consent	12	The patient should share their perspective in one to two paragraphs on the treatment(s) they received	Lines 210-213/Page
	13	Did the patient give informed consent? Please provide if requested	Yes ☑ No ☐
